# Randomized controlled trial of intraosseous access vs. intravenous access in traumatic hemorrhagic shock: Effects on inflammation, hematopoiesis, and coagulation

**DOI:** 10.5937/jomb0-58956

**Published:** 2026-01-06

**Authors:** Gaorong Deng, Lang Jiang, Xin Miao, Yuying Dong, Xiang Gao, Zongfang Li

**Affiliations:** 1 Second Affiliated Hospital of Xi'an Jiaotong University, Department of Orthopaedics, Xian, Shaanxi, 710004, China; 2 Fourth Affiliated Hospital of Nanchang University, Department of Orthopaedics, Nanchang, Jiangxi, 330002, China; 3 Fourth Affiliated Hospital of Nanchang University, Department of Obstetrics and Gynecology, Nanchang, Jiangxi, 330002, China; 4 Second Affiliated Hospital of Xi'an Jiaotong University, National-Local Joint Engineering Research Center of Biodiagnostics and Biotherapy, Xian, Shaanxi, 710004, China

**Keywords:** traumatic hemorrhagic shock, inflammatory mediators, hematopoiesis, coagulation, metabolic disturbances, intraosseous access, traumatski hemoragični sok, inflamatorni medijatori, hematopoeza, koagulacija, metabolički pore-mecaji, intraosealni pristup

## Abstract

**Background:**

This study aimed to evaluate the impact of intraosseous (IO) access on inflammatory mediators, hematopoietic cell function, and coagulation-metabolic disturbances in patients presenting with emergency traumatic hemorrhagic shock (THS), thereby providing clinical evidence to refine IO resuscitation protocols in emergency settings.

**Methods:**

We conducted a randomized controlled trial involving 84 THS patients admitted between February 2024 and February 2025. Participants were allocated equally into two groups: the IO group (n= 42), where vascular access was established via humeral or proximal tibial puncture, and the intravenous (IV) group (n= 42), where conventional peripheral or central venous access was prioritized. Serial measurements were performed at baseline (T0), 24 hours (T1), and 72 hours (T2) post-intervention to assess: (1) inflammatory mediators (IL-1 b, IL-6, IL-10, HMGB1, MDA); (2) hematopoietic parameters (CD34+ cell proportion, CFU-GM /BFU-E colony formation, CXCL12, EPO, and TPO ); (3) coagulation profiles (PT, APTT, and D-dimer); and (4) tissue perfusion indicators (blood lactate and lactate clearance rate). Comparative analyses were conducted both between groups and across different time points.

**Results:**

The IO group demonstrated significantly elevated levels of IL-1P, HMGB1, and MDA at T1 and T2 compared to the IV group (P&lt; 0.05), coupled with reduced IL-10 expression (P&lt; 0.05), indicating exacerbated inflammatory imbalance and oxidative stress. Hematopoietic evaluation revealed progressive declines in CD34+ cell populations, CFU-GM /BFU-E colony formation, and CXCL12 concentration in the IO group at T1 and T2 (P&lt; 0.05), despite modest compensatory increases in EPO and TPO that remained inferior to the IV group (P&lt; 0.05). Coagulation studies showed prolonged PT/APTT (P&lt; 0.01) and higher D-dimer levels (P&lt; 0.05) in the IO group, along with worse blood lactate levels and lactate clearance rates compared to the IV group (P&lt; 0.05), suggesting increased tissue hypoxia and coagulopathy risk.

**Conclusions:**

While IO access enables rapid vascular access for resuscitation and reduces critical intervention time, despite its procedural efficiency in rapid vascular access for resuscitation, IO may inadvertently aggravate systemic inflammatory dysregulation, impair hematopoietic function, and worsen coagulation-metabolic disturbances through mechanisms such as mechanical stimulation, hypothermic fluid infusion, and oxidative stress.

## Abstract (sr)

**Uvod:**

Cilj ove studije bio je da se proceni uticaj intraosealnog (IO) pristupa na inflamatorne medijatore, funkciju hematopoetskih }elija i koagulaciono-metaboli~ke poreme-ćaje kod pacijenata sa hitnim traumatskim hemo ragičnim šokom (THS), čime bi se pružili klinički dokazi za usavrr šavanje IO protokola reanimacije u hitnim slučajevima dijagnozu i predviđanje ishoda.

**Metode:**

Sproveli smo randomizovano kontrolisano ispiti-vanje koje je obuhvatilo 84 pacijenta sa THS primljenih između februara 2024. i februara 2025. godine. Učesnici su podjednako raspoređeni u dve grupe: IO grupu (n=42), gde je vaskularni pristup uspostavljen putem humeralne ili proksimalne tibijalne punkcije, i intravenoznu (IV) grupu (n= 42), gde je konvencionalni periferni ili centralni venski pristup bio prioritetan. Serijska merenja su obavljena na početku (T0), 24 sata (T1) i 72 sata (T2) nakon interven-cije radi procene: (1) inflamatornih medijatora (IL-1 b, IL-6, IL-10, HMGB1, MDA); (2) hematopoetskih parametara (udeo CD34+ celija, formiranje kolonija CFU-G m /BFU-E, CXCL12, EPO i TPO ); (3) profila koagulacije (PT, A PTT i D-dimer); i (4) indikatora perfuzije tkiva (laktat u krvi i brzina klirensa laktata). Komparativne analize su sprove-dene i između grupa i u različitim vremenskim tačkama.

**Rezultati:**

IO grupa je pokazala značajno povisene nivoe IL-1p, HMGB1 i MDA u T1 i T2 u poređenj'u sa IV grupom (P &lt; 0 ,0 5 ), zaj'edno sa smanj'enom ekspresij'om IL-10 (P&lt; 0,05), sto ukazuj'e na pogorsanu inflamatornu ne-ravnotežu i oksidativni stres. Hematopoetska evaluacija je otkrila progresivan pad populacija CD34+ celija, formiranja kolonija CFU-GM/BFU-E i koncentracije CXCL12 u IO grupi u T1 i T2 (P&lt; 0,05), uprkos skromnom kompenzatornom povecanju EPO i TPO koje je ostalo inferiorno u odnosu na IV grupu (P&lt; 0,05). Studije koagulacije su pokazale produženo PT/APTT (P&lt; 0,01) i vise nivoe D-dimera (P&lt; 0,05) u IO grupi, zajedno sa losijim nivoima laktata u krvi i stopama klirensa laktata u poređenju sa IV grupom (P&lt; 0,05), sto ukazuje na povecan rizik od tkivne hipoksije i koagulopatije.

**Zaključak:**

Iako IO pristup omogucava brz vaskularni pristup za reanimaciju i smanjuje vreme kritične intervencije, uprkos svojoj proceduralnoj efikasnosti u brzom vaskularnom pristupu za reanimaciju, IO može nenamerno pogorsati sistemsku inflamatornu disregulaciju, ostetiti hematopoetsku funkciju i pogorsati koagulaciono-metaboličke poremecaje putem mehanizama kao sto su mehanička stimulacija, infuzija hipotermijske tečnosti i oksidativni stres.

## Introduction

Traumatic hemorrhagic shock (THS), a life-threatening condition frequently encountered in emergency medicine, is characterized by massive blood loss leading to reduced effective circulating blood volume, impaired tissue perfusion, cellular metabolic dysfunction, and subsequent organ failure [1]. This complex clinical syndrome, typically resulting from high-energy trauma such as motor vehicle collisions or catastrophic injuries, presents with complex, severe, and urgent conditions [2]. In emergency medicine, establishing rapid vascular access for fluid resuscitation constitutes the cornerstone of THS management [3]. The intraosseous (IO) access offers a unique advantage in circulatory collapse as the medullary venous plexus remains patent, creating a non-collapsible conduit to the central circulation with excellent bioavailability of administered fluids and medications [4]. Current resuscitation guidelines accordingly recommend IO access as the primary alternative when peripheral venous cannulation fails, with subsequent conversion to central venous access once hemodynamic stability is achieved [5]. Numerous studies have validated the procedural efficiency and high success rates of IO access in critical settings, making it the preferred alternative when intravenous (IV) access is difficult, as recommended by international guidelines [6]. Current resuscitation guidelines primarily emphasize the procedural efficiency of IO access (e.g., puncture time, first-attempt success rate) [7] [8], yet lack clinical evidence regarding its systemic impact on the bone marrow microenvironment. This knowledge gap may lead to underestimation of IO's effects on immune homeostasis and hematopoietic recovery, ultimately influencing long-term outcomes. In the era of precision medicine, these conventional metrics, which focus predominantly on technical success and immediate hemodynamic stabilization, present notable limitations. A more comprehensive evaluation should incorporate deeper insights into the underlying pathophysiology of shock, moving beyond simplistic procedural outcomes.

Emerging evidence from basic science research reveals that traumatic insult induces mitochondrial dysfunction in bone marrow mesenchymal stem cells, activating pro-inflammatory cascades through the TLR4/NF- B signaling pathway [9]. Of particular concern is the bone marrow cavity - the central microenvironment for hematopoietic cell differentiation and development - where the potential mechanical or biochemical perturbations induced by direct IO infusion of large volumes of hypothermic fluids or hyperosmotic agents remain systematically uninvestigated. Such disturbances could disrupt local inflammatory mediator release and impair hematopoietic reconstitution, yet this knowledge gap risks clinical underestimation of IO's impact on immune homeostasis recovery and marrow compensatory capacity, ultimately skewing prognostic assessments [10].

To address these critical questions, our prospective study investigates the temporal dynamics of inflammatory mediators during the first 72 hours post-IO access in emergency THS patients, concurrently evaluating functional changes in hematopoietic progenitor cells through colony-forming assays and analyzing alterations in the CXCL12-CXCR4 chemokine axis. By deciphering how IO influences inflammatory networks and hematopoietic recovery in shock states, this research aims to provide molecular biological evidence for refining IO resuscitation protocols.

## Materials and methods

### Study population

Inclusion criteria: (1) Diagnosis of THS; (2) Clinical signs including altered mental status, tachycardia, cold/pale extremities, hypotension, or other perfusion deficits; (3) Shock index (SI) > 1.0 with stage III-IV shock (per ATLS criteria: SBP<90 mmHg, anuria, or GCS<12). Exclusion criteria: (1) Patient/family declined participation; (2) Pregnancy; (3) Fracture or infection at potential access sites; (4) Other forms of shock (e.g., septic, cardiogenic); (5) Injury Severity Score (ISS) (10) <25 to ensure injury homogeneity.

From February 2024 to February 2025, we enrolled 84 consecutive THS patients (55 males and 29 females; mean age 43.55 ± 11.25 years). Injury mechanisms included motor vehicle collisions (n=41), falls from height (n = 15), crush injuries (n = 12), blunt/sharp force injuries (n = 11), and other (n = 5). Written informed consent was obtained from all participants or their legal representatives after full disclosure of study risks. The institutional ethics committee approved the protocol (No. 20240551).

### Study protocol

Patients were randomly allocated to either IV (n=42) or IO (n = 42) groups for IV access and IO access, respectively. Sample size was calculated based on IL-6 differences in pilot data (mean Δ = 15 pg/mL, SD=8.5). With α=0.05, β=0.1, and 20% attrition, 42 patients per group provided 90% power. Randomization was performed using computer-generated random numbers with block randomization (block size=4). Allocation concealment was ensured via sequentially numbered, opaque sealed envelopes opened after patient enrollment. All resuscitations were supervised by senior emergency physicians at or above the attending physician level, and advanced trauma life support was provided for severe trauma patients. Operators (attending-level emergency physicians) received standardized 2-hour training on IO device placement and maintenance prior to study initiation, including instructional videos and hands-on practice.

### Methods

In the IV group, peripheral venous access was initially attempted. If the puncture attempt exceeded 2 minutes without success, central venous access was promptly established. For the IO group, the proximal humerus puncture site was identified at the most prominent aspect of the greater tuberosity, approximately 1-2 cm above the surgical neck. For IO access, puncture site (proximal humerus vs. tibia) was randomly assigned using sealed envelopes to control site-specific confounding. The proximal tibia puncture site was located about 3 cm inferior to the patella and 2 cm medial to the tibial midline on its flat surface. Following standard skin disinfection, the IO needle was advanced perpendicular to the bone until the marrow cavity was entered. The stylet was then withdrawn, and correct placement was confirmed by aspirating bone marrow using a syringe. The needle was then secured, and the IO catheter was flushed with 5-10 mL of 0.9% isotonic saline to maintain patency. Both groups received 30 mL/kg of lactated Ringer's solution at room temperature (22-25°C) infused under 300 mmHg pressure. The intraosseous device was maintained for no more than 24 hours, whereas the central venous line was retained for up to 30 days.

### Laboratory tests

Venous blood samples were collected from patients at 0 hours (T0), 24 hours (T1), and 72 hours (T2) after puncture establishment. The following indicators were analyzed: IL-1β (DLB50), IL-6 (D6050B), IL-10 (D1000B), HMGB1 (IC1690G), CXCL12 (MAB310), EPO (DEPRU0), and TPO (BAF288): These markers were quantified using enzyme-linked immunosorbent assay (ELISA) with commercially available kits (R&D Systems). Malondialdehyde (MDA): Levels were determined via the thiobarbituric acid (TBA) colorimetric method using assay kits (Nanjing Jiancheng Bioengineering Institute). CD34^+^ cell enumeration: Flow cytometric analysis was performed (FACSCanto II, BD Biosciences) using CD34-FITC and CD45-APC antibody staining to identify and quantify CD34^+^ hematopoietic progenitor cells. Compensation was set using UltraComp eBeads^TM^ (Invitrogen), with daily CS&T calibration (BD Biosciences). CD34^+^ gate was defined per ISHAGE protocol. Colony-forming unit assays (CFU-GM) and burst-forming unit-erythroid (BFU-E): Isolated bone marrow mononuclear cells (BMNCs) were cultured in methylcellulose-based semi-solid media (Metho-Cult™, STEMCELL Technologies) supplemented with lineage-specific growth factors (GM-CSF and IL-3 for CFU-GM; EPO and SCF for BFU-E). After 7-14 days of incubation (37°C, 5% CO_2_), colonies containing ≥50 cells were microscopically enumerated. PT, APTT, and D-dimer: These were assessed using an automated coagulation analyzer (Sysmex CS-2500).

Additionally, paired arterial samples were obtained for lactate measurement. Heparinized blood was analyzed via enzymatic electrode methodology (e.g., Radiometer ABL90 blood gas analyzer). The 24-hour lactate clearance rate was computed as: Lactate clearance (%) = (initial l actate-24h l actate)/initial lactatex100.

None of the sample collectors or laboratory technicians knew the patients' groupings.

### Statistical analysis

The data were analyzed using SPSS22.0. Continuous variables are presented as mean ± standard deviation (χ±s) and were compared between groups using independent samples t-tests. Categorical variables were analyzed using chi-square tests or Fisher's exact tests. Longitudinal data were analyzed using repeated-measures ANOVA with post-hoc Bonferroni tests for within-group comparisons, supplemented by independent t-tests for between-group differences at each time point. A *P*-value of less than 0.05 was considered statistically significant.

## Results

### Comparison of baseline characteristics

As shown in Table 1, the two groups did not differ significantly in baseline clinical characteristics, including age, sex, and BMI (*P*>0.05), confirming their comparability. In the IO group, access was established in the proximal humerus in 29 cases and in the proximal tibia in 13 cases, with a first-attempt success rate of 85.71%. In the IV group, 5 patients required a switch from peripheral to central venous access, and the first-attempt success rate was 64.29%. The IO group demonstrated a significantly higher first-attempt success rate than the IV group (*P*<0.05). Additionally, compared with the IV group, the IO group exhibited shorter puncture time, less infusion time, and faster infusion speed (*P*<0.05).

**Table 1:** Comparison of baseline characteristics.

Projects	IV group (n= 42 )	IO group (n= 42 )	t (or χ^2^)	P
Age	43.93 ±11.81	43.17±10.80	0.389	0.759
Sex			0.474	0.491
male	26 (61.90)	29 (69.05)		
female	16 (38.10)	13 (30.95)		
BMI (kg/m^2^)	23.24±3.07	22.57±3.39	0.948	0.346
DBP (mmHg)	77.90±10.40	76.10±9.70	0.825	0.412
SBP (mmHg)	111.43±12.66	112.40±10.47	0.385	0.701
Types of injuries			0.957	0.916
traffic accident	22 (52.38)	19 (45.24)		
fall	8 (19.05)	7 (16.67)		
crush	5 (11.90)	7 (16.67)		
blunt/sharp force injuries	6 (14.29)	5 (11.90)		
Other	1 (2.38)	4 (9.52)		
Puncture site				
proximal humerus	-	29 (69.05)		
proximal tibia	-	13 (30.95)		
First-attempt success	27 (64.29)	36 (85.71)	5.143	0.023
Puncture time (s)	121.07±58.31	85.76±13.34	3.826	<0.001
Infusion time (min)	30.52±2.36	15.76±3.42	23.024	<0.001
Infusion rate (m L/min)	40.29±3.68	122.67±16.00	32.521	<0.001

### Dynamic changes in inflammatory mediators

**Figure 1:**
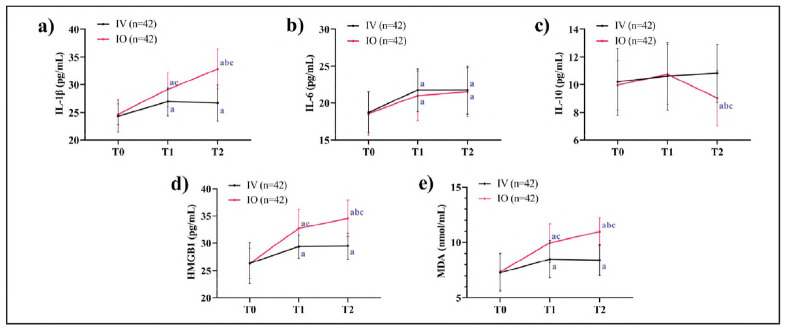
Dynamic changes in inflammatory mediators. a) Comparison of changes in IL-1β, b) Comparison of changes in IL-6, c) Comparison of changes in IL-10, d) Comparison of changes in HMGB1, e) Comparison of changes in MDA. Note: 'a' indicates *P *< 0.05 compared with T0, 'b' indicates comparison with T1, and 'c' indicates P < 0.05 compared with the IV group at the same time.

At T0, no statistical inter-group differences were observed in serum inflammatory mediator levels (*P*>0.05). By T1, the IO group exhibited significantly elevated levels of IL-1β, HMGB1, and MDA compared to the IV group (*P*<0.05), whereas IL-6 and IL-10 levels remained comparable (*P*>0.05). These findings suggest that the IO route may amplify the release of tissue injury-associated mediators and intensify oxidative stress due to localized mechanical stimulation. At T2, the IO group maintained higher expression of HMGB1, IL-1β, and MDA (*P*<0.05), while IL-10 levels were significantly suppressed (*P*<0.05), indicating an inadequate compensatory anti-inflammatory response (Figure 1).

### Impairment of hematopoietic function

**Figure 2:**
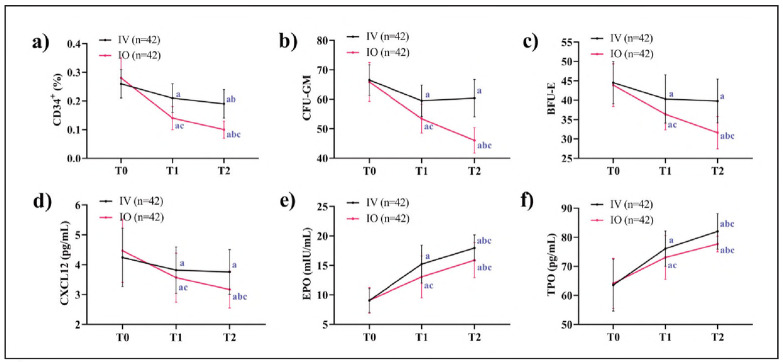
Impairment of hematopoietic function. a) Comparison of changes in CD34^+^, b) Comparison of changes in CFU-GM, c) Comparison of changes in BFU-E, d) Comparison of changes in CXCL12, e) Comparison of changes in EPO, f) Comparison of changes in TPO. Note: 'a' indicates *P *< 0.05 compared with T0, 'b' indicates comparison with T1, and 'c' indicates *P *< 0.05 compared with the IV group at the same time.

Compared to T0, the IO group demonstrated progressive declines in CD34^+^ cells, CFU-GM, BFU-E, and CXCL12 at both T1 and T2, with values significantly lower than those in the IV group (*P*<0.05). This suggests a disruption in the bone marrow hematopoietic niche's chemotactic support capacity. Furthermore, although EPO and TPO levels in the IO group were elevated at T1 and T2, they remained lower than in the IV group (*P*<0.05), likely due to diminished responsiveness of growth factors secondary to localized bone marrow injury (Figure 2).

### Tissue perfusion and metabolic alterations

Blood lactate levels were significantly higher in the IO group than in the IV group at both T1 and T2 (*P*<0.05). Additionally, the 24-hour lactate clearance rate was reduced in the IO group versus the IV group (*P*<0.05), reflecting impaired restoration of tissue perfusion (Table 2).

**Table 2:** Tissue perfusion and metabolic alterations. Note: 'a' indicates P<0.05 compared with T0, 'b' indicates comparison with T1.

Groups	Blood lactate (mmol/L)			24-hour lactate <br>clearance rate (%)
	T0	T1	T2	
IV group (n=42 )	1.32±0.42	0.90±0.08^a^	0.78±0.09^ab^	23.36±29.89
IO group (n 42 )	1.32±0.30	1.11± 0.17^a^	0.87± 0.17^ab^	12.41±22.08
t (or χ^2^)	0.070	7.322	3.116	2.003
* P *	0.944	<0.001	0.003	0.049

### Coagulation dysregulation

**Figure 3:**
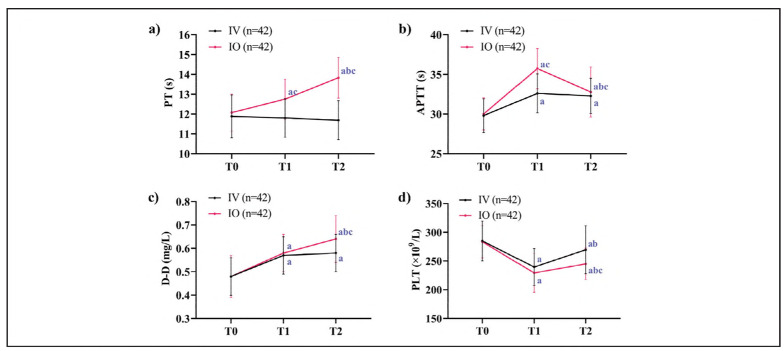
Coagulation dysregulation. a) Comparison of changes in PT, b) Comparison of changes in APTT, c) Comparison of changes in D-D, d) Comparison of changes in PLT. Note: 'a' indicates *P *< 0.05 compared with TO, 'b' indicates comparison with T1, and 'c' indicates *P *< 0.05 compared with the IV group at the same time.

At T1, the IO group displayed prolonged PT and APTT compared to the IV group (*P*<0.01). By T2, PT remained prolonged in the IO group, and D-dimer levels were significantly elevated compared to the IV group (*P*<0.05). Additionally, PLT decreased in both groups at T1 compared to T0 but recovered by T2, though the IO group's PLT remained lower than that of the IV group (*P*<0.05) (Figure 3).

## Discussion

While IO infusion has been increasingly reported in the management of THS, its systemic effects on objective laboratory parameters remain underexplored. Our study comprehensively evaluated the impact of IO access on inflammatory mediators, hematopoietic function, tissue perfusion, and metabolism and coagulation function in THS patients, yielding several key findings:

Regarding inflammatory responses, we demonstrated that IO puncture-induced intramedullary pressure fluctuations and periosteal injury robustly activate inflammatory cascades, particularly elevating IL-1β and HMGB1 levels. This aligns with the mechanistic work by Ibrahim N et al. [11] in animal models, where mechanical stress from marrow puncture was shown to trigger TLR4-dependent IL-iβ secretion. The observed HMGB1 surge is particularly noteworthy, as this damage-associated molecular pattern engages RAGE receptors to amplify systemic inflammation [12] [13]. These results align with previous observations by Deng C et al. [14], who identified a significant association between elevated HMGB1 levels and increased risk of organ failure in trauma populations. Notably, IO infusion of non-warmed fluids may trigger a sudden drop in temperature in the bone marrow microenvironment, leading to mitochondrial dysfunction and excessive reactive oxygen species (ROS) production, evidenced by the characteristic tandem of elevated MDA and depressed SOD activity (oxidative/antioxidant imbalance), collectively exacerbating tissue damage. These findings corroborate experimental data from Stern M et al. [15], where hypothermic fluid resuscitation exacerbated oxidative damage in hemorrhagic shock models through similar mechanisms, manifested by increased MDA and decreased mitochondrial membrane potential. The temporal dynamics of IL-10 regulation presented another critical insight. The marked decline in this anti-inflammatory cytokine at 72 hours post-intervention suggests monocyte functional exhaustion under persistent inflammatory pressure, resulting in an imbalance between pro- and anti-inflammatory networks and creating a »second-hit« effect [16]. This phenomenon provides clinical validation for the »inflammatory exhaustion« hypothesis [Gao Z et al. [17]], demonstrating how sustained inflammatory activation can ultimately deplete the host's capacity to mount appropriate anti-inflammatory responses through cytokine synthesis impairment.

For hematopoietic function, the decline in CXCL12 suggests bone marrow stromal damage impairs hematopoietic stem cell homing, leading to reduced CD34^+^ cells and colony-forming capacity. Additionally, although compensatory increases in EPO and TPO were detected, their upregulation was less pronounced than in the IV group. This attenuated response implies that localized bone marrow injury - potentially mediated by oxidative stress or inflammatory cytokine suppression - may reduce growth factor receptor sensitivity, thereby hindering efficient hematopoietic recovery. Supporting this notion, Asker ME et al. [18] demonstrated in a bone marrow injury model that an inflammatory microenvironment down-regulates EPO receptor expression, blunting erythropoietic responses. Furthermore, the »osmotic shock« hypothesis proposed by Liu Y et al. [19] suggests that hypertonic conditions can induce mitochondrial permeability transition pore opening in hematopoietic stem cells, triggering apoptosis. Given this mechanism, we speculate that hypertonic fluids administered via IO infusion may potentially alter bone marrow interstitial osmolarity, contributing to hematopoietic progenitor cell dysfunction.

Finally, the elevated blood lactate levels and reduced lactate clearance observed in the IO group indicate persistent tissue hypoxia, which may contribute to a self-perpetuating cycle of inflammation and coagulation dysfunction through the following mechanisms: (1) Lactate-mediated hypoxia exacerbation: The accumulation of lactate activates HIF-1α, leading to increased HMGB1 release [20]. HMGB1, in turn, further suppresses mitochondrial respiratory chain activity, worsening hypoxia. (2) Coagulation abnormalities: The prolonged PT/APTT and elevated D-dimer levels suggest concurrent excessive thrombin generation and hyperfibrinolysis in the IO group, which may be driven by IL-6, which promotes tissue factor expression while accelerating platelet consumption. Supporting this, a study by Ueno T et al. demonstrated a significant correlation between IL-6 levels and coagulopathy severity in infectious shock patients [21].

Based on the above findings, we recommend that the following key points be prioritized in future IO procedures: (1) All infused fluids should be prewarmed to 37°C to prevent hypothermia-induced damage to the bone marrow microenvironment. (2) The initial perfusion rate should be carefully regulated (e.g., maintained at 50 mL/min) to minimize mechanical stress on the bone marrow cavity. (3) To mitigate oxidative and inflammatory injury, N-acetyl-cysteine (NAC) should be administered intravenously within 24 hours post-IO resuscitation to counteract MDA-mediated oxidative stress. Alternatively, low-dose corticosteroids (e.g., hydrocortisone at 50 mg/day) or HMGB1 monoclonal antibodies may be considered to suppress excessive inflammatory cascades.

This research has several limitations that should be acknowledged. First, the single-center, small-sample design may limit the statistical power and generalizability of the findings. Second, the 72-hour observation window was insufficient to assess long-term hematopoietic recovery. Third, potential confounding factors, such as the type of resuscitation fluid used, were not evaluated. Fourth, the observed reduction in CXCL12 levels and its association with mechanical IO stimulation require further validation through animal studies or in vitro bone marrow models to establish causality. Future studies should address these limitations to strengthen the evidence base and refine clinical protocols for IO procedures. Fifth, we did not record blood transfusion volumes, surgical intervention timelines, or injury severity scores (e.g., ISS), which may confound inflammatory and coagulation markers.

While IO access exacerbates inflammatory and hematopoietic disturbances, its time advantage in achieving hemodynamic stabilization remains critical when IV access fails. We recommend reserving IO for scenarios where peripheral venous cannulation is impractical, while implementing mitigation strategies (e.g., fluid warming, rate control) to minimize adverse effects.

## Conclusion

Through comprehensive multidimensional monitoring, this study elucidates the dualistic role of IO access in THS resuscitation. The IO approach demonstrates clinically valuable advantages, including high procedural success rates and rapid fluid administration, making it a critical intervention for early hemodynamic stabilization. However, our findings reveal that IO access concurrently potentiates multiple pathological cascades - exacerbating systemic inflammatory dysregulation, impairing hematopoietic recovery, and aggravating coagulation-metabolic disturbances - through mechanistically distinct pathways involving direct mechanical stimulation, hypothermic fluid effects, and oxidative stress-mediated damage. Therefore, when IV access is achievable within 2 minutes, it should be prioritized. If IO is required, mitigation strategies (e.g., fluid warming, rate control) are essential.

## Dodatak

### Consent to publish

All authors gave final approval of the version to be published.

### Availability of data and materials

The data used to support the findings of this study are available from the corresponding author upon request.

### Acknowledgements

Not applicable

### Conflict of interest statement

All the authors declare that they have no conflict of interest in this work.
